# Variations in Concurrent Validity of Two Independent Inertial Measurement Units Compared to Gold Standard for Upper Body Posture during Computerised Device Use

**DOI:** 10.3390/s23156761

**Published:** 2023-07-28

**Authors:** Roger Lee, Riad Akhundov, Carole James, Suzi Edwards, Suzanne J. Snodgrass

**Affiliations:** 1 School of Health Sciences, College of Health, Medicine and Wellbeing, The University of Newcastle, Callaghan, NSW 2308, Australia; 2 Active Living Research Program, Hunter Medical Research Institute, New Lambton Heights, NSW 2305, Australia; 3 Griffith Centre for Biomedical and Rehabilitation Engineering (GCORE), Menzies Health Institute Queensland, Griffith University, Gold Coast, QLD 4222, Australia; 4 School of Health Sciences and Social Work, Griffith University, Gold Coast, QLD 4222, Australia; 5 Sydney School of Health Sciences, Discipline of Occupational Therapy, Faculty of Medicine and Health, University of Sydney, Newcastle, NSW 2308, Australia; 6 School of Health Sciences, Discipline of Exercise & Sport Science, Faculty of Medicine & Health, Sydney University, Sydney, NSW 2006, Australia

**Keywords:** validation, inertial measurement unit, upper body posture, posture, computer use, wearables

## Abstract

Inertial measurement units (IMUs) may provide an objective method for measuring posture during computer use, but research is needed to validate IMUs’ accuracy. We examine the concurrent validity of two different IMU systems in measuring three-dimensional (3D) upper body posture relative to a motion capture system (Mocap) as a potential device to assess postures outside a laboratory environment. We used 3D Mocap and two IMU systems (Wi-Fi and Bluetooth) to capture the upper body posture of twenty-six individuals during three physical computer working conditions (monitor correct, monitor raised, and laptop). Coefficient of determination (R^2^) and root-mean-square error (RMSE) compared IMUs to Mocap. Head/neck segment [HN], upper trunk segment [UTS], and joint angle [HN-UTS] were the primary variables. Wi-Fi IMUs demonstrated high validity for HN and UTS (sagittal plane) and HN-UTS (frontal plane) for all conditions, and for HN rotation movements (both for the monitor correct and monitor raised conditions), others moderate to poor. Bluetooth IMUs for HN, and UTS (sagittal plane) for the monitor correct, laptop, and monitor raised conditions were moderate. Frontal plane movements except UTS (monitor correct and laptop) and all rotation had poor validity. Both IMU systems were affected by gyroscopic drift with sporadic data loss in Bluetooth IMUs. Wi-Fi IMUs had more acceptable accuracy when measuring upper body posture during computer use compared to Mocap, except for trunk rotations. Variation in IMU systems’ performance suggests validation in the task-specific movement(s) is essential.

## 1. Introduction

Computer use is associated with an increased risk of musculoskeletal disorders (MSDs) [1]. As workplace computer-based activities are ubiquitous [2], individuals that are sedentary [3] or engage in poor upper body postures during computer use are more likely to report neck, shoulder, and lower back pain [4]. Musculoskeletal injuries can greatly affect an individual’s daily function, health, and wellbeing [5,6]. Posture-related MSDs have been commonly reported during the COVID-19 pandemic, where many workers utilised ad hoc home office environments [7]. Evaluating upper body postures associated with physical computer working conditions may improve our understanding of the risks of MSDs, to assist in reducing the burden of musculoskeletal injury.

Clinician observation to evaluate their client’s posture is widely practiced [8]; however, it is reliant on a subjective interpretation or perception of the client’s postures during the task. Mechanical measurement devices such as the goniometer, inclinometer, or tape measure offer greater objectivity than observation [9]. However, the reliability of measuring small posture changes (<5°) and the inability to capture dynamic functional movement suggest they only provide a rudimentary kinematic measurement [8,10]. Direct posture measurement systems have high validity and test–retest reliability, e.g., three-dimensional (3D) optical motion capture (Mocap) systems (retro-reflective markers tracked using infrared cameras) [11], 3D electromagnetic systems (magnetic sensors tracked within a magnetic field) [12], and two-dimensional (2D) video analysis (digital video recorded) [13]. Mocap is considered the gold standard for kinematic analysis and can track and record motion accurately; e.g., high discriminative validity was identified for distinguishing differences in the wrist’s range of motion during a computer typing task [14]. Another study of neck postures during computer typing discriminated between individuals with and without chronic neck pain [15]. Limitations of Mocap include time-consuming analysis, specialised software, high initial set-up costs, and the requirement of experienced operators [8,10]. Existing motion capture systems are primarily laboratory-based; thus, generalising 3D motion data to real-world working environments remains challenging [10,16].

An alternative to motion capture for objectively measuring kinematics is the inertial measurement unit (IMU). An IMU is inexpensive ($150.00 to 2500.00 USD per IMU compared to a Mocap system costing $25 K to 500 K USD), portable, and easier for clinicians to operate [17]. IMUs track and record motion in real time with up to nine degrees of freedom [18]. IMUs record 3D motion with internal microelectromechanical sensors (MEMSs) for acceleration (accelerometer), angular velocity (gyroscope), and rotation (magnetometer) [19], with multiple IMUs capable of monitoring multiple body segments simultaneously [20]. As IMUs are not restricted to laboratories [21], this suggests that an individual’s posture can be quantified in their natural environment, which is more likely to capture ‘usual’ working postures compared to the laboratory setting [1]. Evaluating an individual’s ‘usual’ posture during computer use may identify maladaptive postures that are potentially modifiable [22].

IMUs from various manufacturers have shown good reliability [23] and validity [24] in measuring upper body posture during short prescribed tasks [11,25]; e.g., a high coefficient of determination (R^2^ = 0.98) and low error (<1.4°) were reported for a short shoulder validation task within a laboratory environment [26]. IMUs that tracked the upper body movements during a simulated surgical task using a computer demonstrated high concurrent validity compared to Mocap, i.e., neck and trunk flexion/extension angles within 2.9 ± 0.9° (RMSE) and 1.6 ± 1.1° (RMSE), respectively, with excellent agreement for R^2^ < 0.2 [11]. Another study showed IMUs monitoring trunk posture during a seated laptop task demonstrated strong correlations (R^2^ = 0.78) with a mean difference of ~3° compared to Mocap [27]. Field-based studies utilising longer periods of observation for upper body postures have shown acceptable IMU reliability for one hour of computer use [28] and reasonably good sagittal plane accuracy for the upper trunk posture (ranged between 4.1° to 6.6°) during eight hours of dairy parlour monitoring using an accelerometer and gyroscope [29]. However, measurement errors that can dramatically affect device accuracy will most likely occur as the duration of the monitoring period increases, e.g., sensor shifting initial placement [30], magnetic interference inducing gyroscopic drift [29], and wireless connection issues or mishandling an IMU device during use [31]. One major limitation for an IMU is gyroscopic drift (orientation measurement error within the gyroscope) resulting from data noise, though algorithmic filters can mitigate these accumulative errors [19,20]. Further, IMUs may record data ‘on board’, or stream data via Wi-Fi or Bluetooth, and each of these may have effects on the data that are collected. Previous research had identified that validation of an IMU is dependent on the specific task performed, the selected post-processing algorithm, and the environment [10], though the majority of studies do not clearly specify these factors. Despite this fundamental importance, validation data are rarely disclosed [17,32,33], which may explain the lack of IMU acceptability into routine clinical practice [25,34].

As IMU technology is relatively new, the limitations of IMU technology have not been thoroughly explored, especially in the context of measuring upper body posture, which requires extended data collection over long time periods to simulate postures that happen when people work on their computers for extended periods. Thus, further research to validate IMUs during computer use is paramount before they can be recommended for use in posture monitoring and, consequently, clinical decision making to mediate postural MSD in real-world environments. Therefore, we aim to assess the concurrent validity of two different commercially available IMU systems compared to a 3D motion capture system to measure upper body postures associated with three physical computer working conditions within a simulated laboratory environment.

## 2. Materials and Methods

This cross-sectional observational study quantified IMU validity for 3D positioning of IMU instrumentation, and IMU validity for upper body posture of individuals during computer use within a laboratory environment. IMU instrumentation position and upper body posture were measured using a 3D Mocap system (Qualisys AB, Gothenburg, Sweden) and two types of inertial measurement units, MMR-MetaMotionR (Mbientlab Inc., San Francisco, CA, USA) and Biscuit (WithRobot., Seoul, Republic of Korea), during three different computer working conditions. Upper body kinematics were used to determine IMU validity (i.e., accuracy) for both IMU instrumentation position and body segment position and joint angle compared to the 3D Mocap system.

Ethical approval was granted from The University of Newcastle Human Research committee (H-2018-0232). All participants provided written informed consent prior to data collection.

### 2.1. Participant Recruitment

To appropriately evaluate validity when measuring upper body posture with IMUs, human participants were required. Recruitment methods for participants included electronic noticeboards for students and staff, and flyers posted throughout the University of Newcastle and surrounding area. Participants were screened for eligibility by telephone. Eligible participants were between 18 and 55 years of age and were required to regularly use computers. Participant demographics included gender, age, occupation, body mass (mechanical floor scale Model 762, Seca, Hamburg, Germany) and height (standardised stadiometer), average time spent using any computerised device in a single day, and self-reported frequency of device use.

### 2.2. Task

This study required participants to perform a 15 min computerised typing task using ‘their own work’ repeated across three different computer working conditions: desktop monitor set at correct height (monitor correct), desktop monitor set too high (monitor raised), and laptop (specifications in next section). Participants’ ‘own work’ was defined as typing work they would typically complete during computer use. Participants brought in their own work to simulate their real-world typing task. A two-minute warm up or familiarisation period was conducted prior to each 15 min working condition and a 5 min rest during the condition changeover period was used post-condition [35]. Participants were required to stand up to have a break from their sitting posture between conditions. After standing, they had five minutes to stand, walk, or sit at their own discretion within the laboratory. The order of each condition was randomised to reduce any effects from task fatigue. Each participant completed their three conditions on a single day within a single session.

### 2.3. Electronic Devices and Experimental Condition

Two of the three physical computer working conditions (monitor correct and raised) utilised a 23-inch (53.3 cm) LCD monitor (Model No. P2314H, Dell, Inc., Round Rock, TX, USA) attached to a sliding mount (Kangaroo Pro., Ergonomic Essentials, North Ryde, Australia) that was projected from the Dell Latitude laptop in a closed lid position. A standard 104-key Windows keyboard (Model No. KB212B) and corded optic mouse (Model No. MS116) was used for both the monitor correct and monitor raised conditions. For monitor correct, height was set at 1/3 (33%) above the participants’ horizontal eye level [36]; for monitor raised, the height was adjusted to 150 mm above the monitor correct position. The laptop condition utilised a 15.6-inch (39.6 cm) Dell Latitude E6540 laptop (Dell Inc., Round Rock, USA) with no external mouse or keyboard. Participants were allowed to self-adjust the laptop screen tilt to suit personal preference during the warm-up period, as minor adjustments in screen tilt do not affect posture [37]. Fluorescent lighting similar to a typical office environment provided adequate lighting with no monitor glare present as per guidelines [38]. All working conditions used a standard-height office desk (760 mm) with dimensions of 1200 × 600, with table position verified prior to each participant by floor markings and a height and lumbar adjustable office chair (Model No. QU26, Sturdy Framac, Padstow, NSW, Australia). Participants were advised to wear their ‘usual’ clothing and corrective eyewear if required. Participants were ergonomically adjusted (standardised) as per guidelines [38] prior to each condition and reminded to self-adjust into their usual working postures. Distance between participant and monitor or laptop screen was adjusted to arm’s length with approximately 90°. Participants were not required to perform any functional or prescribed movements during any condition.

### 2.4. Data Collection and Body Landmarks

Nine Qualisys infrared cameras (Oqus 300+; approximate size of data collection volume 2.3 m^2^) tracked and recorded 3D movement of the retro-reflective body markers at a sampling rate of 100 Hz using Qualisys Track Manager (QTM) software (Version 2022.1, Qualisys AB, Gothenburg, Sweden). High accuracy for joint angle analysis had previously been demonstrated using the Qualisys 3D motion system [39,40,41]. Calibrating the Mocap prior to each participant session ensured each camera received a relative 3D reference in association with the other cameras [42]. Motion capture for upper body segments were defined by 52 retro-reflective markers comprehensively described in a previous study [35].

This study utilised two types of commercially manufactured IMU systems: (1) MMR-MetaMotionR, which used Bluetooth for real-time data transfer or on-board data storage (8 MB); and (2) Biscuit, which used Wi-Fi. All IMUs and Mocap simultaneously recorded motion data during all conditions. Sampling rates for Bluetooth IMUs were 100 Hz and Wi-Fi IMUs 10 Hz. Sampling rates of 10 Hz and above are recommended to prevent violation of Nyquist theorem and loss of kinematic data during sedentary working tasks [43]. Wi-Fi IMUs required a custom-built code to stream real-time Wi-Fi data through a standard router to a dedicated laptop operating MATLAB software (R2019a MathWorks Inc., Natick, MA, USA). Bluetooth IMUs used low-energy Bluetooth to send real-time data from each Bluetooth IMU to proprietary software (Mbientlab Inc, MetaBase application) on an iPad Tablet (Apple Inc., Cupertino, CA, USA) as duration in data collection far exceeded the on-board 8 MB storage capacity. Close proximity between sensors and iPad (within 2 metres) ensured high Bluetooth signal strength.

This study utilised quaternion data (Qx, Qy, Qz, and Qw) to represent rotations with greater numerical stability compared to Euler angles, which may encounter gimbal lock and result in difficulties interpreting the rotational data [44]. Fused quaternion IMU data represented sensor orientation, i.e., absolute roll, pitch, and yaw. All IMUs logged date and time (milliseconds).

Both IMU systems used a BOSCH BNO055 chip, with an on-board sensor fusion algorithm (BSX Lite Fusion library, Bosch Sensortec GmbH, Reutlingen, Germany), which incorporated an offset calibration for influences in magnetic distortion related to the magnetometer, drift associated with the gyroscope, sensor calibration, and a Kalman filter [45] (Bosch Sensortec, 2015). Manual Wi-Fi IMUs’ sensor calibration was completed prior to each participant with Bluetooth IMUs initiated via the MetaBase application.

**Figure 1:**
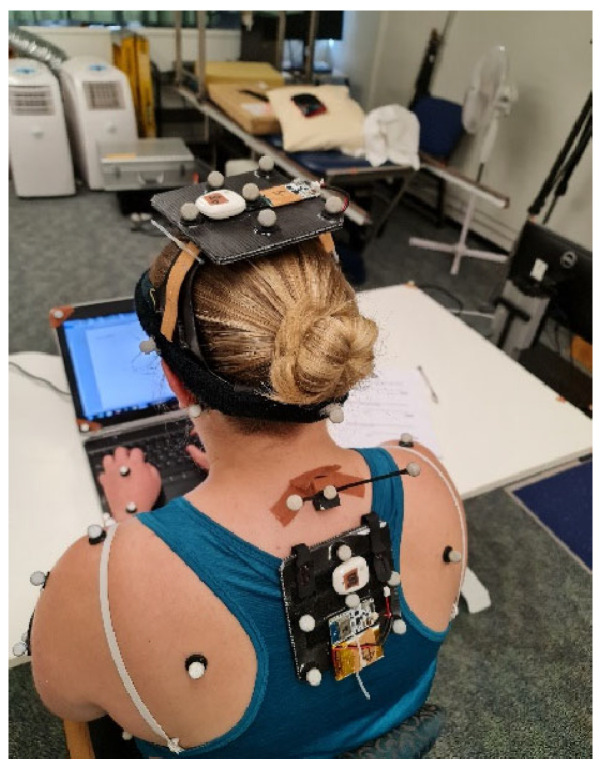
IMU rigid plates and retro-reflective marker placement for head and upper trunk segments.

Both the Bluetooth and Wi-Fi IMUs were adhered to rigid plates with six retro-reflective markers per plate (Figure 1) to enable tracking of movement for the IMUs themselves through the Mocap, one on the head and another on the upper trunk. The head rigid plate was attached using an adjustable headband, with the upper trunk rigid plate attached onto a skin-tight singlet to attenuate any skin artefact (Figure 1). The position of the trunk IMU was over T4, located by palpation while the participant was seated.

Both IMU systems were calibrated and initialised to start recording prior to the Mocap recordings. Participants were required to perform a static anatomical pose for 20 s prior to any dynamic motion trial for each condition. Participants performed three large head nods in the sagittal plane during the 1st minute at the initial dynamic recording during each condition. A total of 14 min was used for analysis due to the 1st minute being omitted to remove the prescribed movements (head nods) from influencing the data. IMUs were synchronised using the first maximum peak value in the primary movement direction for all trials. All data were collected during typical working hours (between 9 to 5) to represent typical office hours.

### 2.5. Motion Capture System Kinematic Classification, Analysis, and Reduction

Cartesian local co-ordinate system was as follows: x-axis = mediolateral axis (frontal plane: lateral flexion), y-axis = anterior–posterior axis (sagittal plane: flexion/extension), and z-axis = superior–inferior axis (transverse plane: rotation). The joint co-ordinate system standard proposed by Cole et al. [46] was followed and a x, y, z Cardan sequence of rotation was used to express the intersegmental joint angles.

The Mocap modelling for the inertial properties when classifying the head, neck, thorax, and upper trunk was created using geometric primitives [47]. As applied in previous studies [35,48], the reduction and analysis for 3D geometric primitives for the upper trunk segment [UT] was measured relative to the laboratory co-ordinate system (UTS). This was defined as the midpoint between bilateral acromioclavicular joint markers (cephalad end) and bilateral bottom rib markers (caudad end) with the radius of each segment ends being 50% of the distance between these markers. Upper trunk segment angle was calculated as the thorax relative to the laboratory co-ordinate system to gauge the degree of forward body lean. The head–neck segment [HN] (combination of both head and neck segments) measured relative to the laboratory co-ordinate system was defined by the mid-head (cephalad end) and an imaginary line between the 7th cervical vertebrae (C7) and sternal notch (SN) (caudad end). The radius of these head–neck landmarks was 50% of the distance between the lateral headband markers. The head–neck segment relative to the upper trunk segment [HN-UT] joint angle was formed by the rotational difference between the HN and UTS. Both IMU types mounted onto the head rigid plate were used to define the head segment relative to the laboratory co-ordinate system for each IMU, and similarly for IMUs quantifying the upper trunk segment relative to the laboratory system. Prior to calculation of individual joint kinematics, all raw kinematic co-ordinates were filtered using fourth-order zero lag Butterworth digital low-pass filter (fc = 6 Hz) to attenuate noise [49]. Three-dimensional kinematic data were exported to MATLAB using Visual 3D software (Version 6; C-Motion, Germantown, MD, USA).

### 2.6. Data Processing

Raw data from the Biscuit IMU collected at 10 Hz were upsampled to 100 Hz using MATLAB’s resample function to enable statistical comparisons with the Wi-Fi IMUs and Mocap systems that were collected at 100 Hz. Orientation for each IMU was aligned to the Mocap local co-ordinate system [46]. Additionally, all IMU angle data was unwrapped using MATLAB’s unwrap function to prevent wrapping around ±180°, thus enabling greater visualisation of any potential gyroscopic drift.

### 2.7. Statistical Validation Analysis

IMU orientation relative to the laboratory co-ordinate system for HN and UTS and HN-HTS for each separate physical working computer condition (monitor correct, monitor raised, and laptop) were each separately compared to Mocap. The coefficient of determination (R^2^) was used to quantify the strength of the relationship between the IMU time-series and the corresponding ground truth (Mocap) time-series. Specifically, R^2^ is a goodness of fit measure of how much of the total variance in the Mocap time-series is explained by the IMU time-series. This provides a measure of the similarity of the two time-series, with a higher R^2^ value indicating the time-series are more similar. Additionally, the root-mean-square error (RMSE) was used to quantify the average magnitude of the error (in degrees) between the IMU time-series and the corresponding Mocap time-series. Combining both R^2^ and RMSE provides a comprehensive overview of the IMU signal quality and, consequently, validity. Additionally, to assess if the IMUs maintained the strength of the relationship compared to Mocap, R^2^ and RMSE were analysed during the first working condition following recording at the 2nd-minute mark (60 s) and repeated for the 14th minute (60 s). Any non-uniformed data (i.e., data with missing data points across the 100 Hz sampled time-series) were resampled, using MATLAB’s resample function, to a uniform rate of 100 Hz using the timestamps associated with each datapoint. Validity was considered high if R^2^ > 0.75, moderate if R^2^ 0.4–0.74, and poor if R^2^ < 0.39 [23]. All analysis used MATLAB (version 2019a, Mathworks, Natick, MA, USA).

## 3. Results

Twenty-six participants (14 female, 12 male; mean ± standard deviation age: 26.7 ± 10.2 years; body mass: 77.5 ± 18.7 kg; and height: 173.8 ± 9.2 cm) completed this study. The majority of participants were university students (*n* = 16/26, 61.5%); others were university staff (*n* = 4/26, 15.4%) or held other occupations (*n* = 6/26, 23.1%) (Table 1).

Wi-Fi IMUs for sagittal plane movements demonstrated high validity (R^2^ >0.75) for HN and UTS segments and moderate validity (R^2^ 0.4–0.74) for joint angles during all conditions (Table 2). For frontal plane movements, Wi-Fi IMUs showed high validity for joint angles and moderate validity for HS and UTS segments across all conditions. Some conditions demonstrated high validity for rotation movements (HN during monitor correct and raised) with others being moderate and poor (Table 2). Bluetooth IMUs had moderate validity (R^2^ 0.4–0.74) for sagittal movements for segments (HN and UTS during monitor correct and laptop conditions) and for UTS (monitor raised); frontal plane movements had moderate validity for UTS (monitor correct and laptop), with the other condition being poor. The lowest validity values were for Bluetooth IMUs during rotation movements (R^2^ <0.24 across all conditions; Table 2). An analysis comparing data taken shortly following recording (2nd minute of first task) and data from the 14th minute revealed that the Wi-Fi IMUs maintained validity for both time-series compared to Mocap, with the Bluetooth IMUs demonstrating lower validity values at the 14th minute (Table 3). Non-uniform raw data originated from the Bluetooth IMUs due to sporadic data loss.

## 4. Discussion

The purpose of this study was to evaluate the concurrent validity of two different commercially available IMU systems compared to Mocap in measuring 3D upper body postures (HN, UTS, and HN-UTS) across three physical computer working conditions within a simulated laboratory environment. This study considered R^2^ as the primary statistic to evaluate validity, i.e., the strength in linear relationship of the IMU compared to Mocap, with the significance in RSME dependent on the quality of R^2^. Overall, the Wi-Fi IMUs demonstrated greater accuracy than the Bluetooth IMUs when compared to the gold-standard Mocap across all conditions (Table 2). Wi-Fi IMUs showed high validity (R^2^ > 0.75) for sagittal plane movements for both segments (HN and UTS), in contrast to moderate and low validity for the Bluetooth IMUs during all conditions. Wi-Fi IMUs showed high validity for the joint angle (HN-UTS) for frontal plane movements during each condition, compared to the Bluetooth IMUs demonstrating poor validity. Bluetooth IMUs’ performance was compromised by sporadic data loss and the lack of a calibration status. There were potential magnetic disturbances inducing gyroscopic drift predominantly for rotational movements for both Bluetooth IMUs and the upper trunk Wi-Fi IMU across all conditions; e.g., the Wi-Fi IMU for UTS showed poor validity (R^2^ < 0.20), while Bluetooth IMUs’ validity was poor for all segments and joint angles (R^2^ between 0.08 to 0.24 across all conditions; Table 2). The subsequent devaluation of R^2^ values for joint angles resulted from the increased magnitude of orientation estimation errors associated with rotational movements. A separate time-series analysis (i.e., data from the 2nd and 14th minute of time-series) identified that the Wi-Fi IMUs maintained the strength in the relationship compared to Mocap across these data points, with Bluetooth IMUs showing a mostly low data relationship (Table 3). These findings suggest variations in performance between IMU systems is evident, meaning that some IMU systems may not be suitable for some types of testing protocols or environments. In the current study, the Bluetooth IMUs failed to accurately measure upper body posture during computer use in the laboratory environment. Despite the Wi-Fi IMUs demonstrating high concurrent validity for most joint angles, research to validate IMUs for specific measurement procedures and within specific environments is required to ensure device accuracy prior using IMU data to interpret research outcomes.

IMU accuracy compared to Mocap for measuring upper body posture has been previously reported, e.g., neck segment (sagittal plane) within an RSME of 2.9° and upper trunk within an RSME of 1.6° during a simulated surgical task [11] lasting <5 min. Another laboratory study using ten cycles (approximately 1 min) of upper trunk movements in the sagittal plane resulted in an RMSE of between 4.1° and 6.6° [29]. A third study measuring head and upper trunk posture during sagittal plane movements during a short 7 m walking task reported IMU R^2^ values of 0.82 and 0.58, respectively, within an RMSE of 3° [50]. Despite the differences in tasks, duration in motion capture, and IMU hardware between these studies, the accuracy of our Wi-Fi IMUs was comparable during all conditions over the 14 min time-series; e.g., head segment R^2^ ranged between 0.91 to 0.94 for within an RMSE of <2.42° and upper trunk between 0.82 to 0.85 with RMSE of <1.45°. In contrast, Bluetooth IMUs for sagittal plane movements had a moderate to poor validity for the head segment (0.33 to 0.58 and within <6.90°), and upper trunk (0.49 to 0.63 and within <4.30°), and may be better suited for tracking posture during a shorter time-series. The ability for an IMU to track small changes in upper body movement is important. Previously reported neck and upper trunk postures most likely to cause MSD during computer use (sagittal plane) were >−6.5° [51] and >−5° [52] (neutral position being 0°), respectively. Thus, the sensitivity within an IMU to detect subtle differences i.e., <5° in movement behaviour, is essential [11,50,53].

**Figure 2:**
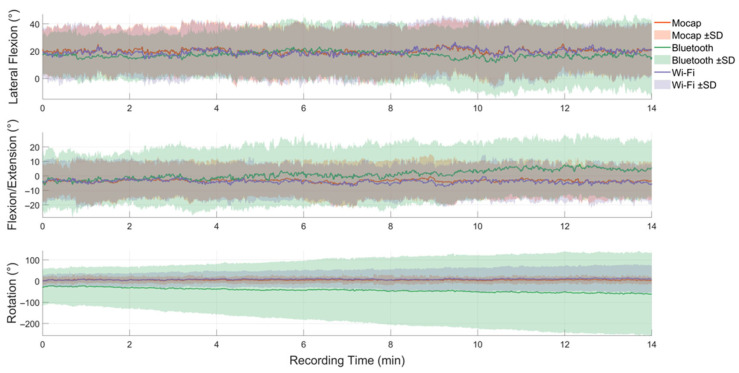
Upper trunk movement (averages) for twenty-six participants showing the relationship between the Wi-Fi IMU, Bluetooth IMU, and motion capture (Mocap) data across 14 min of typing during the first condition (included monitor correct, monitor raised, or laptop) across three axes with standard deviation denoted by shaded areas. Note that for flexion/extension and lateral flexion, the Wifi IMUs throughout the time-series provide data that is consistent with motion capture, with drift for the Bluetooth IMU after approximately the 8th minute for lateral flexion and approximately the 4th minute for flexion/extension. For rotation, the Wifi IMU provides similar data to motion capture, but the Bluetooth IMU displays significant offset at time zero and drift.

**Figure 3:**
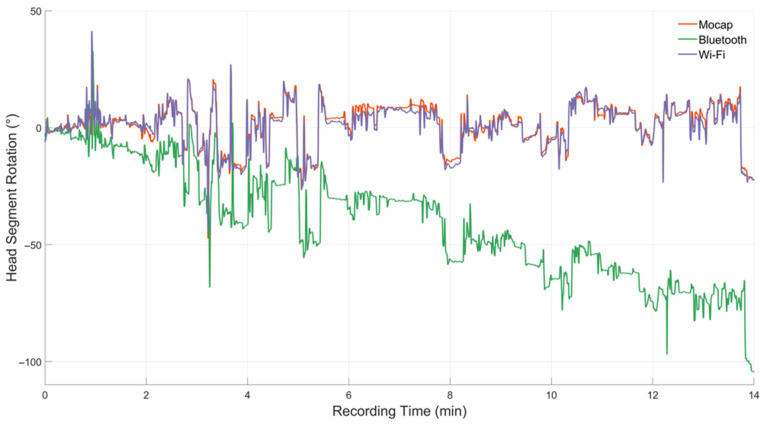
Rotation movement for HN segment from one participant during typing for 14 min demonstrating the magnitude of gyroscopic drift for the Bluetooth IMU compared to the Wi-Fi IMU and motion capture.

In this current study, differences in performance between the two IMU systems in measuring upper body posture was identified. Sporadic loss of data and gyroscopic drift within the Bluetooth IMUs produced a moderate but mostly poor data relationship across all axes compared to Mocap (during each condition) (Figure 2). Rotational movement accuracy for the Wi-Fi IMUs were also affected by gyroscopic drift. Known measurement errors such as skin artefact can degrade kinematic tracking as the IMU may shift from the underlying anatomical landmark during movements such as walking, running, or jumping [54,55]. However, in this study, we assume these errors were negligible from the low intensity of movements per task (typing) and method of attachment of the IMU and Mocap retro-reflective markers to a solid plate, meaning each sensor type would experience an identical movement, as the mounting plate and the sensor attached move en bloc. Potential interference of the Bluetooth signal within the laboratory environment may have contributed to the sporadic loss in data, despite the close proximity of the iPad tablet (within 2 m) from each participant to maintain signal strength. Errors in orientation estimates occurring from gyroscopic drift can originate from fluctuating offset averages and noise within the data that produces accumulative measurement errors over time, which can greatly affect accuracy [56,57]. Use of a magnetometer (as in this study) may compensate for gyroscopic drift [56]; however, magnetic field disturbances which are typically present indoors can induce errors [58]. In this study, rotational movements for both Wi-Fi and Bluetooth IMUs were affected by magnetic disturbance inducing some degree of gyroscopic drift during all conditions (Table 2), with the largest drift errors associated with both Bluetooth IMUs (Figure 2). A previous study investigating upper body movement also reported gyroscopic drift using the Bluetooth IMU [59]. Our separate analysis using two time-series (2nd and 14th minute of data) of data identified poor R^2^ values for the Bluetooth IMUs at the 14th minute of operation, suggesting the Bluetooth IMUs data continually deviated for rotation movements across the time-series when compared to Mocap (Figure 3), and this would likely worsen over longer periods of data capture. Wi-Fi IMUs’ data remained relatively stable for rotation movements throughout these time periods (2nd and 14th minute data points; Table 3), though the upper trunk Wi-Fi IMU for rotation movements across the entire time-series demonstrated gyroscopic drift that resulted in poor validity for UTS and, subsequently, reduced the reported accuracy for these joint angles across each condition (Table 2). This may suggest smaller samples of data (<1 min) may be inadequate for detecting these subtle changes in gyroscope drift over time for some IMUs. Magnetic disturbance from the office chair backrest (metal support beneath cushion) or magnetism within the laboratory may have influenced greater gyroscopic drift as shown during rotational movements for the upper trunk segment for both Wi-Fi and Bluetooth IMUs, and, potentially, magnetic disturbance may explain the poor R^2^ values shown for the Bluetooth IMU head segment (Table 2 and Table 3).

Commonly, algorithmic filters such as a Kalman filter are designed to integrate all MEMS to mitigate errors in gyroscopic drift and improve overall orientation estimates [45,60,61]. In this study, both IMU systems utilised Kalman filters, though variations in IMU system performance may be affected by the appropriate application of filter parameters. Despite the Bluetooth IMUs having no acknowledgment of calibration status within their software application, all instructional prompts were followed during the Bluetooth IMUs’ device set-up. However, the proprietary software application and filter-processing quality are unknown. Capturing postures during computer use remains challenging due to the nature of movements during each task (i.e., small movements and little variation in movement) [28]. Posture monitoring over long time periods using multiple time-points is recommended, as relatively short time periods (<2 min) of consecutive sampling may contribute to loss of movement precision [62]. Collecting data over longer time periods may affect IMU performance [63], as greater gyroscopic drift in this current study was associated with continuous data captured (Figure 2). A possible method to mitigate gyroscopic drift may be to capture data at multiple time-points by repeatedly re-calibrating the IMU to reset the orientations estimates. However, this may interrupt the participant during their task and prevent capturing postures over longer typing periods. Therefore, further work is required in advancing the technological methods to reduce gyroscopic drift to achieve accuracy in rotational movements.

### Strengths and Limitations

To our knowledge, this is the first study to assess two commercially available IMU systems compared to a 3D motion capture system during three different working conditions using computers. Wi-Fi IMUs had acceptable accuracy in quantifying upper body posture. Despite the greater duration in motion capture reported in this study compared to other studies, the repeatability of these results is unknown due to participants attending a single laboratory session. Participants in this study presented their ‘usual’ postures during computer use in contrast to large deliberate non-typical movements that are prescribed in most studies [11,30,59]. Due to individuals in this study demonstrating their natural movements when typing during each condition, greater variability in the movement data between participants is likely across the entire 14 min time-series. The Bluetooth sensor streamed data at a sampling rate of 100 Hz, whereas the Wi-Fi sensor sampled at 10 Hz. This may be one reason for the greater data loss from the Bluetooth sensor, despite the close proximity between sensors and iPad (within 2 m) to ensure high Bluetooth signal strength. Nevertheless, 10 Hz was adequate to prevent a violation of the Nyquist theorem [43] as demonstrated by the Wi-Fi IMU data having acceptable accuracy (error) in evaluating upper body posture. Upsampling data using an interpolation filter may have a small negative effect on the Wi-Fi IMU signal quality, that may lower the R^2^ and increase the RMSE. However, this well-established processing method [30,64] is expected to have a minimal effect on our comparisons. Larger-than-expected gyroscopic drift errors occurred for rotational movements for the upper trunk Wi-Fi IMU and for both Bluetooth IMUs. These may potentially relate to the longer time of data collection compared to other studies, and possible magnetic disturbances within the vicinity of the IMUs to metals within a computer workstation environment. In addition, the Bluetooth IMUs’ application of the proprietary filter and sporadic loss of Bluetooth signal may contribute to errors compared to Mocap. Despite efforts to minimise the size of the rigid plates worn by participants, the size of the apparatus worn may have affected the postures participants demonstrated in the study. This study did not evaluate user comfort and device wearability. Further work is required to determine an IMU’s clinical suitability, and whether it can distinguish upper body movement behaviour between healthy individuals and individuals experiencing MSDs. Additionally, IMU reliability testing is required to ensure consistency in the reported results.

## 5. Conclusions

This study comprehensively assessed the concurrent validity of two commercially available IMU systems for measuring upper body postures during three different computer working conditions relative to a Mocap system within a simulated laboratory environment. Our findings suggest the Wi-Fi IMU is an acceptable device to accurately track upper body postures during a continuous time-series, except for trunk rotations where accuracy was moderate to poor, potentially due to magnetic disturbances. Sporadic loss in data, lack of calibration status, and gyroscopic drift may have induced large movement errors for Bluetooth IMUs, suggesting they are unacceptable for measuring upper body posture during computer use. Despite an IMU being a more accessible device compared to Mocap for assessing upper body postures during computerised device use, the presence of differences between the accuracy of IMU systems compared to the gold-standard motion capture for task-specific movement(s) suggests prior validation is essential before interpreting research outcomes.

## 6. Practical Considerations

These findings suggest researchers need to validate their IMU systems prior to reporting their findings. Clinicians should ensure that manufacturers report the validation for the specific tasks they wish to measure. The mode of IMU data transfer (Wi-Fi, Bluetooth, or on-board storage) must be appropriate to suit the application and duration of movement tracking. Rotational measurements using IMUs should be viewed with caution, as an unspecified magnitude of gyroscopic drift is inherent in all systems. Rotational drift that occurred in this study progressively caused signal deviation from the Mocap which, over longer time periods, will greatly affect IMU accuracy. Algorithms and filters to mitigate drift and magnetic interference are not typically reported by manufacturers. Future research should investigate IMU validity and reliability testing to ensure consistency in the reported outcomes and clinical suitability.

## Figures and Tables

**Table 1:** Participant characteristics.

Characteristic	(*n* = 26)
Age (yr), mean (SD)	26.7 (10.2)
Sex: Female *n* (%) Male *n* (%)	14 (54) 12 (46)
Body mass (kg), mean (SD)	77.5 (18.7)
Height (cm), mean (SD)	173.8 (9.2)
Participant reporting daily device use *n* (%):	
Desktop sitting	21 (81.0)
Desktop standing	4 (15.0)
Laptop	16 (61.5)
Tablet	7 (27.0)
Phone	24 (92.0)
Self-reported length of time of device daily use (hours), mean (SD) ^a^:	
Desktop sitting	4.15 (1.5)
Desktop standing	0.14 (0.3)
Laptop	4.24 (3.2)
Tablet	1.13 (0.5)
Phone	3.92 (2.2)
Occupation: University student University staff Non-university participants	14 4 6

^a^ Average time of individuals that used each device.

**Table 2:** R^2^ and RSME comparing IMU systems to motion capture for 3D segment and joint angles during 14 min of typing under three conditions (monitor correct, monitor raised, and laptop).

			Monitor Correct			Monitor Raised			Laptop		
			HN	UTS	HN-UTS	HN	UTS	HN-UTS	HN	UTS	HN-UTS
Wi-Fi IMU System	R^2^ (±SD)	x	0.52 (0.21)	0.62 (28.7)	0.77 (0.2)	0.53 (32.1)	0.70 (0.2)	0.75 (29.1)	0.65 (0.2)	0.60 (44.8)	0.81 (0.2)
		y	0.91 (0.2)	0.82 (1.7)	0.52 (0.2)	0.94 (1.2)	0.85 (0.1)	0.53 (0.3)	0.94 (0.2)	0.84 (2.0)	0.54 (0.2)
		z	0.80 (0.2)	0.13 (8.8)	0.53 (0.3)	0.84 (3.8)	0.12 (0.1)	0.35 (9.0)	0.54 (0.2)	0.20 (8.9)	0.36 (0.2)
	RMSE (±SD)	x	3.19 (0.2)	0.95 (9.1)	3.92 (0.3)	2.31 (3.8)	0.76 (0.1)	4.17 (9.1)	2.21 (0.2)	0.98 (9.1)	3.16 (0.2)
		y	2.42 (0.2)	1.02 (30.1)	4.60 (0.9)	1.78 (32.6)	0.98 (3.8)	5.12 (30.9)	1.67 (0.4)	1.45 (45.7)	4.43 (0.8)
		z	4.65 (0.2)	11.19 (1.7)	13.04 (1.3)	2.69 (1.2)	12.46 (2.5)	13.59 (26.2)	7.10 (0.9)	8.90 (2.7)	12.62 (1.1)
			HN	UTS	HN-UTS	HN	UTS	HN-UTS	HN	UTS	HN-UTS
Bluetooth IMU System	R^2^ (±SD)	x	0.38 (0.2)	0.46 (1.8)	0.11 (0.2)	0.18 (1.1)	0.37 (0.1)	0.10 (24.0)	0.26 (0.2)	0.42 (1.7)	0.16 (0.2)
		y	0.54 (0.9)	0.63 (2.7)	0.10 (0.3)	0.33 (1.1)	0.49 (0.1)	0.06 (4.9)	0.58 (0.2)	0.58 (3.3)	0.07 (0.2)
		z	0.14 (0.2)	0.16 (27.4)	0.13 (0.2)	0.14 (30.2)	0.22 (0.2)	0.10 (28.4)	0.09 (0.2)	0.24 (42.3)	0.08 (0.2)
	RMSE (±SD)	x	3.91 (0.2)	1.09 (28.0)	16.90 (0.8)	3.46 (30.2)	1.50 (0.2)	17.26 (28.4)	3.63 (0.2)	1.53 (42.7)	11.22 (0.2)
		y	6.00 (0.2)	1.46 (1.7)	10.91 (0.2)	6.90 (1.1)	4.30 (0.1)	10.86 (24.3)	5.03 (0.2)	3.08 (1.8)	9.93 (0.2)
		z	42.93 (0.2)	17.56 (8.2)	43.27 (0.3)	42.90 (3.6)	15.99 (0.1)	58.45 (8.2)	37.32 (0.2)	10.88 (8.3)	38.32 (0.2)
Range of validity:											
	High if R^2^ > 0.75				Moderate if R^2^ 0.4–0.74				Poor R^2^ < 0.39		

R^2^, coefficient of determination; RMSE, root-mean-square error in degrees; SD, standard deviation. X axis = frontal plane, lateral flexion; Y axis = sagittal plane, flexion/extension; Z axis = transverse plane, rotation.

**Table 3:** R^2^ and RSME comparing IMU systems to motion capture for 3D segments and joint angles during the 2nd and 14th minute of typing during the first condition undertaken at commencement of IMUs being initiated (includes monitor correct, monitor raised, or laptop).

			2nd Minute			14th Minute		
			HN	UTS	HN-UTS	HN	UTS	HN-UTS
Wi-Fi IMU System	R^2^ (±SD)	x	0.73 (0.3)	0.70 (0.3)	0.74 (0.3)	0.58 (0.3)	0.66 (0.3)	0.71 (0.4)
		y	0.71 (0.4)	0.63 (0.3)	0.82 (0.2)	0.92 (0.2)	0.78 (0.2)	0.59 (0.3)
		z	0.81 (0.3)	0.50 (0.3)	0.89 (0.1)	0.82 (0.2)	0.50 (0.4)	0.72 (0.3)
	RMSE (±SD)	x	4.38 (3.9)	0.56 (0.3)	4.01 (3.7)	1.43 (0.9)	0.46 (0.3)	2.07 (1.4)
		y	3.69 (2.7)	0.84 (0.5)	3.30 (3.3)	1.20 (0.9)	0.56 (0.3)	2.11 (1.5)
		z	4.35 (5.0)	1.30 (1.0)	3.54 (3.1)	2.34 (3.0)	0.95 (0.5)	2.71 (2.5)
			HN	UTS	HN-UTS	HN	UTS	HN-UTS
Bluetooth IMU System	R^2^ (±SD)	x	0.64 (0.3)	0.44 (0.3)	0.27 (0.3)	0.15 (0.2)	0.30 (0.3)	0.16 (0.1)
		y	0.58 (0.3)	0.45 (0.3)	0.31 (0.4)	0.19 (0.1)	0.43 (0.3)	0.12 (0.2)
		z	0.54 (0.4)	0.28 (0.3)	0.52 (0.4)	0.12 (0.1)	0.41 (0.3)	0.13 (0.1)
	RMSE (±SD)	x	5.21 (4.2)	0.88 (0.6)	16.22 (25.7)	2.53 (1.3)	0.73 (0.5)	7.36 (13.9)
		y	4.67 (2.6)	0.97 (0.5)	9.89 (6.5)	4.74 (2.5)	1.12 (0.9)	3.77 (2.0)
		z	16.08 (21.5)	2.73 (3.5)	16.03 (21.3)	5.66 (3.6)	1.10 (0.7)	5.81 (3.6)
**Range of validity:**								
	High if R^2^ > 0.75			Moderate if R^2^ 0.4–0.74			Poor R^2^ < 0.39	

R^2^, coefficient of determination; RMSE, root-mean-square error in degrees; SD, standard deviation. X axis = frontal plane, lateral flexion; Y axis = sagittal plane, flexion/extension; Z axis = transverse plane, rotation.

## Data Availability

Not applicable.
